# Anodizing of Hydrogenated Titanium and Zirconium Films

**DOI:** 10.3390/ma14247490

**Published:** 2021-12-07

**Authors:** Alexander Poznyak, Andrei Pligovka, Marco Salerno

**Affiliations:** 1 Department of Electronic Technology and Engineering, Belarusian State University of Informatics and Radioelectronics, 6 Brovki Str., 220013 Minsk, Belarus; poznyak@bsuir.by; 2 Research and Development Laboratory 4.10 “Nanotechnologies”, Belarusian State University of Informatics and Radioelectronics, 6 Brovki Str., 220013 Minsk, Belarus; 3 Department of Micro- and Nanoelectronics, Belarusian State University of Informatics and Radioelectronics, 6 Brovki Str., 220013 Minsk, Belarus; 4 Department of Functional Materials and Hydrogen Technology, Military University of Technology, 2 Ka-liskiego Str., 00-908 Warsaw, Poland; marco.salerno@wat.edu.pl

**Keywords:** anodizing, TiO_2_, ZrO_2_, titanium oxide, zirconium oxide, valve metal, titanium hydride, zirconium hydride, Ti:H, Zr:H

## Abstract

Magnetron-sputtered thin films of titanium and zirconium, with a thickness of 150 nm, were hydrogenated at atmospheric pressure and a temperature of 703 K, then anodized in boric, oxalic, and tartaric acid aqueous solutions, in potentiostatic, galvanostatic, potentiodynamic, and combined modes. A study of the thickness distribution of the elements in fully anodized hydrogenated zirconium samples, using Auger electron spectroscopy, indicates the formation of zirconia. The voltage- and current-time responses of hydrogenated titanium anodizing were investigated. In this work, fundamental possibility and some process features of anodizing hydrogenated metals were demonstrated. In the case of potentiodynamic anodizing at 0.6 M tartaric acid, the increase in titanium hydrogenation time, from 30 to 90 min, leads to a decrease in the charge of the oxidizing hydrogenated metal at an anodic voltage sweep rate of 0.2 V·s^−1^. An anodic voltage sweep rate in the range of 0.05–0.5 V·s^−1^, with a hydrogenation time of 60 min, increases the anodizing efficiency (charge reduction for the complete oxidation of the hydrogenated metal). The detected radical differences in the time responses and decreased efficiency of the anodic process during the anodizing of the hydrogenated thin films, compared to pure metals, are explained by the presence of hydrogen in the composition of the samples and the increased contribution of side processes, due to the possible features of the formed oxide morphologies.

## 1. Introduction

Anodic oxide films of aluminum (Al) and other valve metals (Ta, Nb, Ti, Hf, Zr, and W) are promising materials for electronic technology [1,2,3,4,5,6]; therefore, electrochemical behavior was investigated [7,8,9,10,11,12,13,14,15,16,17,18,19,20,21]. At the same time, under certain conditions, all these metals (Al [22], Ta [23], Nb [24], Ti [25], Hf [26], Zr [27], W [28], and others [29]) absorb significant amounts of hydrogen, and the volume of the absorbed hydrogen is several orders of magnitude larger than the volume of the metal. Metal hydrides have being studied intensively for a long time, both from a fundamental point of view [29,30,31,32,33,34,35,36,37,38] and for various applications. They can be used, for example, for storing hydrogen, as shown in the case of Al [39,40,41], Ti [42,43], Zr [44,45,46], and alloys [47]. In addition, hydrogenated metals are currently used in the manufacturing of catalytic [48] and photocatalytic materials [49,50,51], super capacitors [52], microwave absorbers [53], and electronic products [54]. The absorption of significant amounts of hydrogen strongly alters the properties of the starting material. The metal becomes brittle [55,56] and, upon further saturation and under certain conditions, it completely collapses, turning into a powdery hydride, which is easily removed from the substrate surface by a stream of dried air, which requires the development of stabilization processes. A numerous number of works are devoted to the electrochemical behavior of titanium [12,13,17,18,19,20] and zirconium [21,57,58]; however, only a few of them are indicated here. There are known work devoted to the study of the hydrogen evolution reaction and the absorption of hydrogen into titanium during its cathodic treatment [59,60,61,62,63,64,65,66,67,68,69,70,71,72,73,74,75,76] and without potential application [77]. One study [78] deals with the electrochemical properties of hydride–proton conductor interfaces at room temperature, in order to ascertain their degree of reversibility. In another study [79], the cathodic reduction behavior of the anodic oxide film on titanium, and the hydrogen absorption behavior, have been investigated.

However, the analysis of literature sources allows to conclude that the behavior of the hydrogenated thin films of valve metals during electrochemical anodizing has not been studied yet. Short reports on the possibility of electrochemical anodic oxidation of hydrogenated titanium and zirconium appeared for the first time in 1999 [80,81]. There are a number of works that are relatively close on the topic [82,83,84,85,86,87], in which the effect of titanium hydride on the formation of Nanoporous TiO_2_ on Ti, during anodizing, has been investigated. Titanium hydrides were formed after cathodizing, profoundly impacting the formation of Nanoporous TiO_2_ on Ti by anodizing. However, in some studies [82,83], the process of the anodic oxidation of a thin surface layer of hydrogenated titanium on the surface of a titanium sample is poorly characterized. In the titanium and the hydrogen-charged Ti in the deaerated 5 M NaOH at 303 K, anodic polarization curves are shown. It is obvious from the indicated dependences that the densities of the currents flowing through the hydrogenated samples during anodic polarization are significantly higher than in the case of pure titanium. These studies are of an applied nature and a narrow focus. At the same time, the use of metal hydrides is very diverse. The methods of hydrogenation are numerous and are not limited to cathodic saturation of the evolved hydrogen; furthermore, the electrochemical behavior of metal hydrides during their electrochemical anodic oxidation in various electrolytes and modes is interesting, both from a fundamental point of view and in connection with the prospects for multiple applications.

This work presents the results of Ti and Zr hydrogenated thin film anodizing. The work reveals, firstly, the establishment of the fundamental possibility of the anodizing of thin-film hydrogenated zirconium and titanium in acid aqueous solutions; secondly, the character of voltage- and current-time responses; thirdly, the study of the impact of hydrogenation degree, the nature of electrolyte and anodizing conditions on the oxidation process.

## 2. Materials and Methods

In the experiments, Zr and Ti, with a thickness of 150 nm, were deposited by magnetron sputtering of 99.95% targets on silicon wafer (p-type 111, 3”, 381 ± 15 μm thick, 8.5–11.5 Ω·cm, Wacker Chemie AG, Munich, Germany). The deposition chamber was initially evacuated to 5 × 10^−7^ mbar, with subsequent sputtering using 99.998% argon at 5 × 10^−3^ mbar. In the mode of discharge power stabilization, the discharge power was maintained at a level of 0.9 kW. In this case, the discharge current was approximately 3.0 A and the discharge voltage varied from 430 to 560 V. Then, the silicon wafer with the sputtered metal was cut; one part of it was used for hydrogenation, and the other (non-hydrogenated)part was used as a reference sample for subsequent anodizing and analysis. Hydrogenation of thin metal films was carried out at atmospheric pressure in a stream of H_2_ at a temperature of 703 K within 30, 40, 60 and 90 min in a single-zone multi-pipe diffusion system CД.OM-3/100 (Union Technology, Zelenograd, Moscow, Russia).

Acids for anodizing were supplied by the Belaquilion (Minsk, Belarus) additional-liability company and manufactured by Sigma-Aldrich, Inc. (Darmstadt, Germany). A programmable power supply 5751 A (Keysight Technologies Inc., Santa Rosa, CA, USA) was used as the anodizing unit, controlled by a personal computer (PC) with homemade software written in LabVIEW. Programmable digital multimeters 34470 A (Keysight Technologies Inc., Santa Rosa, CA, USA) were used to record the voltage-time responses, controlled by a PC with R&D Lab 4.10 developed software. Anodizing of hydrogenated and non-hydrogenated films was carried out with constant stirring in the following:

(1) 1% wt. solutions of boric (BA) and tartaric acid (TA) in combined mode;
(2) 0.6 M solution of TA in galvanostatic mode;
(3) 0.6 M solution of oxalic acid (OA) and TA in potentiodynamic mode.

In the combined mode, firstly, anodizing was carried out in potentiodynamic mode. Then, upon reaching a stationary voltage, the process passed into potentiostatic mode. Anodizing was carried out in a specialized PTFE electrochemical cell [88] with a horizontal sample and an Al cathode. The area of the anodized surface of each sample was 1.54 cm^2^. The initial anodizing temperature was 293 K, and the increase in anodizing temperature during the entire process did not exceed 2 K.

The analysis of literature sources showed that the anodizing of hydrogenated thin films of titanium and zirconium had not previously been carried out; therefore, it was not possible to make the choice of electrolytes on the basis of previous studies. Therefore, electrolytes were selected according to two criteria. Firstly, the possibility of titanium and zirconium activation as a result of hydrogenation was taken into account; therefore, the authors tried to avoid the chemical aggression of electrolytes, leaving only the electrochemical component. The least aggressive electrolytes were selected. Secondly, electrolytes were chosen whose behavior was well known to the authors and the study of which had traditionally been carried out for several decades in the laboratory.

The depth distribution of general elements within the films was obtained with the help of Auger electron spectroscopy (AES) using a PHI-660 Auger microprobe (Perkin Elmer Inc., Waltham, MA, USA) equipped with a LaB_6_ electron emitter, cylindrical mirror analyzer and a differentially pumped Ar^+^ ion sputter gun with step-by-step sputtering of the specimen’s surface layer with an energy of 5 keV of argon ions in an ultra-high vacuum (<10^−9^ torr). Primary electron beam potential and current were 5 kV and 10 nA, respectively. Diameter of electron beam was less than 200 nm, with the depth of penetration into the film being about 2 nm. The intensities of Auger signals for all chemical elements were registered in the energy region 30–1500 eV.

## 3. Results and Discussion

### 3.1. Anodizing in Boric Acid

**Figure 1:**
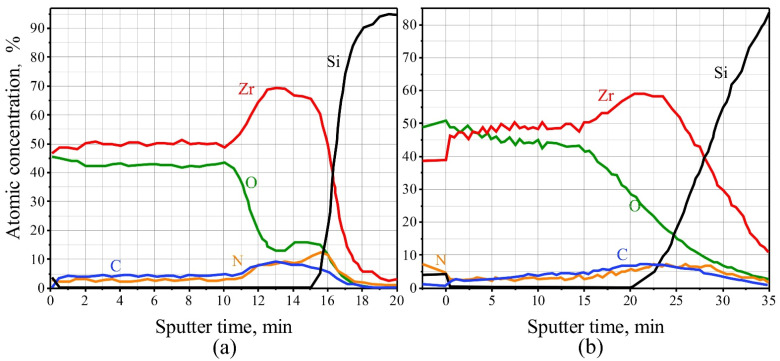
Auger electronic profile of the distribution of elements in a hydrogenated zirconium film after anodizing in a 1 wt.% solution of boric (**a**) and 1 wt.% tartaric (**b**) acid. The hydrogenation time was 30 min.

BA is the least aggressive acid electrolyte for anodizing [89,90], in which barrier oxide films are formed [91]. During the anodizing of hydrogenated Ti and Zr samples in a 1 wt.% aqueous solution of BA in a combined mode, it turned out that the anodizing current-time response resembled the typical profile for barrier oxide layer formation, the current flowing through the cell remained small, the process proceeded slowly, and it was almost impossible to distinguish characteristic stages thereof. At the same time, it was also impossible to determine, from the time response, when the anodizing process ended. The fact that a Zr valve metal oxide is formed in the anodizing process is proven by the AES profile of the distribution of the main and impurity elements over the oxidized metal thickness, as shown in Figure 1a. The Zr anodic oxide was obtained by anodizing in a 1 wt.% BA solution in a combined mode (firstly, in potentiodynamic mode, with a potential growth rate of 1 V·s^−1^ and a maximum reached potential of 300 V; then, holding in the potentiostatic mode (for 10 min) of hydrogenated Zr (hydrogenation time: 30 min).

### 3.2. Anodizing in Oxalic Acid

Attempts to anodize samples of hydrogenated titanium and zirconium in 0.6 M OA showed that, at anodic voltage sweep rates of the order of a few V·s^−1^, violent gas evolution occurred, a current of the order of 100 mA flew through the cell, and the anodizing process ended too quickly to conduct a detailed study. A significant increase in the current, in comparison with the anodizing of a pure metal, was also noted by other researchers [82,83], as well as by us when anodizing hydrogenated samples in other electrolytes (see below), but in the discussed case, the currents were extreme. The authors suggest that high currents and intense gas evolution can be caused by side processes. These can be the decomposition of water, accompanied by the release of molecular oxygen (Equation (1)) and/or the oxidation of the anion of oxalic acid (Equation (2)), leading to the release of carbon dioxide.

(1) $${2H}_{2}O-4e=4H^{+}+O_{2}↑,$$

(2) $$C_{2}O_{4}^{2-}-2e={2\mathrm{CO}}_{2}↑$$

The latter assumption is supported by the fact that none of the other experiments displayed such a catastrophically rapid completion of the process. It is likely that the nanostructured surface of oxides formed as a result of the anodizing of hydrogenated metals [82,83,84,85,86,87], effectively catalyzing the anodic oxidation of the oxalate anion.

### 3.3. Anodizing in Tartaric Acid

**Figure 2:**
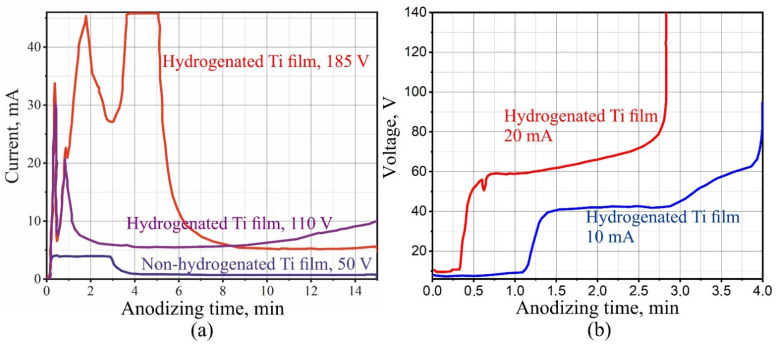
Anodizing time responses of (**a**) combined (sweep rate 1 V·s^−1^ up to 50, 110 and 185 V) in 1% tartaric acid of non-hydrogenated, hydrogenated titanium films and (**b**) galvanostatic (direct current 10 and 20 mA) in 0.6 M tartaric acid of hydrogenated titanium films.

The optimal electrolyte for studying the anodizing of Ti and Zr hydrides from all the used electrolytes turned out to be the aqueous solution of TA. The combined anodizing time response of both non-hydrogenated and hydrogenated Ti deposited on silicon wafers in a 1% TA solution is shown in Figure 2a. It can be observed that the profile for anodizing non-hydrogenated Ti corresponds to the descriptions for the cases of obtaining a barrier oxide layer on Al and other valve metals [7,92,93,94,95,96,97]. At the same time, the anodizing time response of the hydrogenated samples significantly differed from the reference sample. Firstly, despite the fact that the potential sweep rate in all three cases was the same (1.0 V·s^−1^), the maximum anodizing current differed significantly. This indicates that significantly higher electrical conductivity of the anodic oxide formed in the case of the anodizing of the hydrogenated samples. Secondly, the current during the anodizing of the hydrogenated samples changed in a more complex manner than in the case of pure Ti.

A visual observation showed that, during the anodizing of the hydrogenated samples, for several tens of seconds, the formation of anodic oxide of Ti (AOTi) was similar to the anodizing of the non-hydrogenated samples, but after the first minute, the hydrogenated samples became cloudy, and upon further anodizing, they were anodized at a higher potential of 185 V (Figure 2a), and complete destruction and removal of AOTi, in the form of a finely dispersed powder, occurred. At the same time, destruction of the film was not observed for the sample anodized to a voltage of 110 V. However, the authors suggest that with longer anodizing of the sample, complete destruction of the film would also occur.

Having anodized hydrogenated Zr samples, destruction did not occur; the AES profile of the distribution of elements over the thickness of one of the anodized samples is shown in Figure 1b. An anodic Zr oxide was obtained by anodizing a 30 min hydrogenated Zr sample in a 1% TA solution in a combined mode (with a potential growth rate of 1.0 V·s^−1^ up to 300 V, and subsequent holding in potentiostatic mode for 13 min).

During galvanostatic anodizing of the valve metal, as the barrier anodic oxide grows, there should be a linear increase in the anodic voltage [91,94,95,96,97]. The galvanostatic anodizing voltage-time responses at 10 and 20 mA, and at a current of 0.6 M TA, of Ti samples hydrogenated within 40 min are shown in Figure 2b. It appears that during the anodizing of hydrogenated Ti, the nature of the voltage variation over time differs from that of non-hydrogenated Ti [94], in that one may observe the presence of two sections with different voltage growth rates. Figure 2b shows that the voltage sweep rate depends on the current density, which is also typical for the galvanostatic anodizing of non-hydrogenated valve metals.

**Figure 3:**
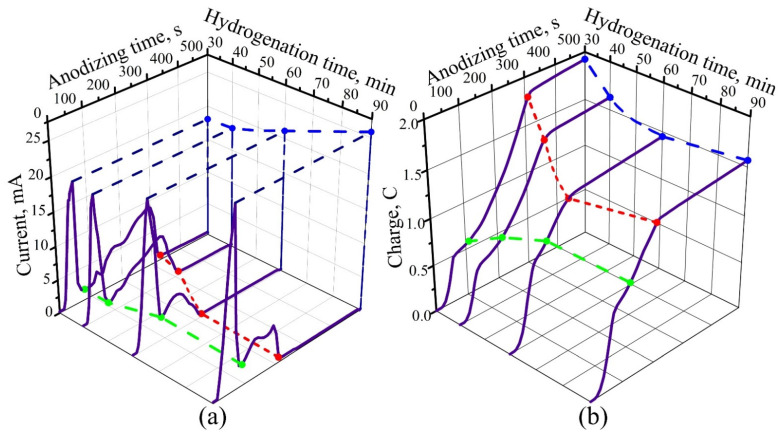
(**a**) Time responses and (**b**) time dependences of the charge during potentiodynamic anodizing of titanium films of various degrees of hydrogenation in 0.6 M tartaric acid. The voltage sweep rate during anodizing was 0.2 V·s^−1^ for all samples.

The investigation of anodized hydrogenated Ti sample features in the potentiodynamic mode showed that the time response of potentiodynamic anodizing depends, to a significant extent, on the time of sample exposure in a hydrogen atmosphere, with all the other conditions being equal. A family of such curves, taken for an anodic voltage sweep rate of 0.2 V·s^−1^ for the case of anodizing in 0.6 M TA, is shown in Figure 3. These curves also show the time dependences of the charge consumed for the anodizing of differently hydrogenated Ti. The anodizing time was 500 s for all the samples.

The obtained time response curves, which also radically differ from those for pure Ti, are characterized by the presence of two clearly pronounced maxima The first is “sharp” and high, followed by a “bell-shape”, which is lower and stretched. It turned out that the time response of potentiodynamic anodizing depends, to a significant extent, on the time of holding the sample in a hydrogen atmosphere, with all the other conditions being equal. An increase in the holding time of the samples in a hydrogen stream (and, consequently, an increase in the degree of hydrogenation) leads to a decrease in the time of complete oxidation of the film. The shape and ratio of the main peaks on the time response curve also change as follows: the maximum value of anodic current (the first, “sharp” peak) increases, while, on the contrary, the height of the second, “bell-shaped” maximum decreases. It is especially interesting that a threefold increase in the duration of holding the samples in a hydrogen atmosphere leads, contrary to expectations, to a 25% decrease in the charge consumed for film oxidation. However, the charge corresponding to the first peak still increases. This allows to assume that the first peak corresponds to the electrochemical transformations of hydrogen in the Ti sample, and the second peak corresponds to the actual oxidation of Ti and the production of AOTi.

**Figure 4:**
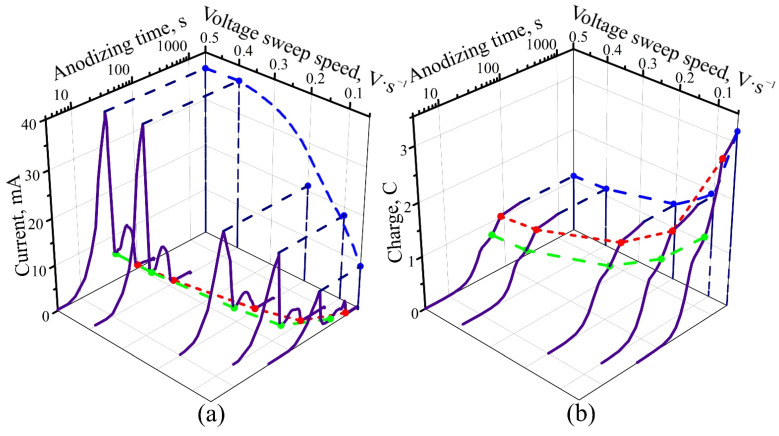
(**a**) Time response and time dependence (**b**) of the charge for potentiodynamic anodizing of hydrogenated titanium films in 0.6 M tartaric acid, at different rates of voltage growth. The hydrogenation time was 60 min for all samples.

The voltage sweep rate also affects the nature of the anodizing process. A hydrogenated Ti sample with a holding time in a hydrogen current of 1 h was selected, and its various fragments were anodized in the potentiodynamic mode, with anodic voltage growth rates in the range of 0.05–0.5 V·s^−1^. The anodizing process was stopped when the voltage reached 100 V. The time dependence of the current and charge consumed for oxidation of the hydrogenated Ti are shown in Figure 4. A tenfold increase in $\frac{dU}{dt}$ leads to an increase in the maximum value of anodic current by about four times; in this case, the charge consumed for complete oxidation of the film is reduced by a factor of three. Such a strong influence of electrical modes on the efficiency of the anodizing process was observed here for the first time, to the best of our knowledge.

It should be noted that, during the anodizing of hydrogenated titanium samples in 0.6 M TA in the potentiodynamic mode, the samples did not fail in any of the cases, and the visually anodized hydrogenated samples did not differ in any way from the anodic oxide of non-hydrogenated titanium. At the same time, during the anodizing of hydrogenated samples, a much higher charge occurred than during the anodizing of pure titanium. Differences in the time responses of anodizing can be due to several reasons, such as the following:

(1) The change in the conductivity of the film formed during anodizing of Ti:H.
(2) The emergence of the current due to the oxidation of atomic hydrogen, distributed in the volume of the metal.
(3) The occurrence of a larger number of side reactions catalyzed by titanium dioxide, formed from hydrogenated samples.

In addition, the structure of the anodic oxide formed as a result of the anodizing of hydrogenated titanium may turn out to be nanoporous [82,83,84,85,86,87], which can stimulate the consumption of electricity for a large number of side processes. This is possible due to the high catalytic activity of titanium dioxide. Significant differences in the initial samples, hydrogenation techniques, electrolyte composition, and electrical modes of anodic oxidation do not allow for a correct comparison of the results considered in the above-mentioned works with the results of the studies presented in the proposed article. At the same time, this work is a continuation of research [80,81], and further development of this topic for scientific and applied interest is expected.

## 4. Conclusions

The study of the anodizing behavior and the composition of hydrogenated samples of valve metals, using Ti and Zr models with a thickness of 150 nm as examples, showed that, as a result of anodizing, anodic oxides of the studied metals are formed. The study of the distribution of elements over the thickness in completely anodized aqueous solutions of hydrogenated zirconium samples with boric and tartaric acids, using Auger electron spectroscopy, indicates the formation of zirconia. The nature of the anodizing of pure and hydrogenated Ti is radically different. The process of potentiodynamic anodizing of hydrogenated Ti at 0.6 M tartaric acid is clearly divided into two stages. The current-time response with potentiodynamic anodizing of hydrogenated Ti in 0.6 M tartaric acid is characterized by the presence of a high, “sharp” peak in the first stage, followed by an extended “bell-shaped” maximum in the second stage. The increase in the holding time of Ti, from 30 to 90 min, in a hydrogen stream, at the same sweep rate of the anodic voltage equal to 0.2 V·s^−1^, leads to a reduction in the anodizing time and in the charge spent on complete implementation of the oxidation process. However, at the same time, this leads to the increase in the charge corresponding to the first, “sharp” maximum and to the radical decrease in the charge corresponding to the second, “bell-shaped” maximum. The efficiency of potentiodynamic anodizing decreases significantly with the decreasing anodic voltage sweep rate from 0.5 to 0.05 V·s^−1^ for samples held in a hydrogen atmosphere for 60 min.

## Data Availability

Not applicable.
